# Social representations of malaria in a southern malian community: an ethnographic qualitative study

**DOI:** 10.1186/s12936-022-04298-0

**Published:** 2022-09-29

**Authors:** Bourema Sissoko, Mohamed Yunus Rafiq, Jiaqi Rosemary Wang, N’bamori dite Naba Sissoko

**Affiliations:** 1 Institute of Anthropology, School of Social Development, East China Normal University, Minhang District, 500 Dongchuan Road, Shanghai, 200241 China; 2 Faculty of Foreign Languages, Southwest Forestry University, 296 Bailongsi, Kunming, Yunnan 650224 China; 3 Department of Social Sciences, New York University Shanghai, 1555 Century Avenue, Pudong New District, Shanghai, 200122 China; 4 School of Pediatrics, Nanjing Medical University, Nanjing, China

**Keywords:** Ethnography, Malaria, Perceptions, Social representations, Therapeutic routes, Mali

## Abstract

**Background:**

Malaria is one of the prime reasons for medical consultation and the major cause of morbidity and mortality in Mali. To assess and understand the dynamics of social representations of malaria, the anthropological research was conducted in the Wayerema II neighbourhood of the health district of Sikasso, southern Mali.

**Methods:**

This was an ethnographic study conducted qualitatively in 2011 and 2016 through informal conversations, 70 semi-structured interviews, and participant observations with key actors. The observations, conversations, and interviews investigated local people’s perceptions and knowledge about malaria, and how and to what extent the cultural and popular representations of the disease can have an impact on therapeutic routes.

**Results:**

Mosquitoes are the principal agent of the transmission of malaria. However, the ubiquitous yet casually-claimed aetiological agents, causative, nosographic entities differ from—although sometimes integrated into—the biomedical dimension. For example, some communities perceive *Kono*, a complicated and pernicious form of malaria that often occurs among children, to originate from a supernatural force. “Bird disease” is another term used for *Kono* in Mali and other West African countries. Thus, overall, *Kono* is defined through the entanglements with cultural factors, namely the idiosyncratic habits, customs, and beliefs of the population of Wayerema II neighbourhood in the health district of Sikasso, Southern Mali. Wayerema II residents particularly tend to link therapeutic recourse amongst the afflicted not only to biomedical models but to sociocultural and popular perceptions and representations of malaria.

**Conclusion:**

In the findings, self-medication through both traditional and modern medical techniques was the most frequent therapeutic modality. Hence, the integration of local popular knowledge with the biomedical register can contribute to a comprehensive understanding of social representations and perceptions of malaria, and qualitative improvements in the malaria control programme.

## Background

Malaria is an endemic disease, which is predominant in the tropical zones of sub-Saharan Africa. In 2020, malaria was reported with an estimated 241 million cases and 627,000 deaths worldwide, with most cases occurring in the World Health Organization (WHO) African Region (greater than 90%). The WHO African Region bears a disproportionately high share of the global malaria burden. In 2020 this region was home to 95% of all malaria and 96% of malaria mortality (deaths). Children under five years of age accounted for about 80% of all malaria deaths in the Region. In the Sahel sub-region of Africa, most childhood malaria mortality and morbidity occur during the rainy season [1–3].

In Mali, malaria is the primary reason for medical consultation during the season of high transmission (June–October). According to the *Système Local d’Information Sanitaire (SLIS)* 2018, health facilities recorded 2,345,481 malaria cases for the entire year [4]. Malaria was the trigger for a high number of deaths in 2018, with 1178 documented [4].

With the statistical data in mind, in Mali and following the example of other African and Central American countries, it is certain that socio-cultural representations and other popular aetiologies are determinants in the use of health care [5–8]. Moreover, Tieman Diarra examines the role of representations in shaping the choices about therapists and therapeutic itineraries in the Bankoni neighbourhood, Commune I of the district of Bamako, Mali, arguing that conceptual understandings of the disease would considerably result in differences in people’s medical choice, especially between conventional and biomedical treatment: “some [patients] started with traditional therapy and ended up with biomedicine, others alternate between them or even resort to both medicines simultaneously” [9].

Indeed, it is believed that representations play an important role in framing practices and may pose very profound influences on a societal level. Social representations are used to explain that recourse would be essentially determined by the financial situation of patients and/or their families, and they can also be used to explain and understand recourse and therapeutic routes. Anne Luxereau focuses on the situation in Maradi, Niger, and states: “people’s ideas about health and illness remain prevalent in shaping or guiding their choices, and determine therapeutic itineraries” [10].

It is under this context that this work has been undertaken to assess the community’s perceptions of malaria in the Wayerema II area in the health district of Sikasso and analyse how the socio-cultural representations of disease can affect the therapeutic routes taken by malaria patients. The results of this study can also contribute to an understanding of the people’s knowledge, attitudes, practices, and beliefs about malaria for improving the National Malaria Control Programmes (NMCP) in Sikasso and Mali.

Structurally, this article first discusses the research methodology, then the social and modern representations of malaria, the socio-economic impacts, and finally the therapeutic itineraries of the patients and/or their families.

## Methods

### Study area

This article is based on fieldwork on social representations of malaria in the neighbourhood of Wayerema II, in the health district of Sikasso, Sikasso region, southern Mali. According to the 2009 general census, Sikasso is the second-largest Malian city, with 225,753 residents. Its initial area, which is 28,530 square-kilometres (km), comprises 15 neighbourhoods, referred to as “quartiers”. The municipality of Sikasso is the capital of Sikasso region, the third administrative region of Mali; Sikasso region is also a crossroads between Mali, Ivory Coast, Guinea, Burkina Faso, and Togo.

Located in the Sudanese zone, Sikasso experiences a tropical Sudanese climate characterized by rainfall varying from 1300 to 1500 millimetres per year with a prevalence of 62%. This level of rainfall makes Sikasso prone to malaria. The region covers an area of 76,480 km^2^ which is 5.7% of the national territory with a density of 31 inhabitants per km^2^. Accessibility to health care is 58% within a radius of 5 km and 80% within a radius of 15 km [11, 12].

### Study design

It also applied a descriptive, qualitative, and inductive design. This study was approached with an ethnographic fieldwork focus, which used observation participants, informal conversations, and semi-structured interviews related to key informants and/or actors. This fieldwork in the health district of Sikasso was complemented by bibliographical research of the perceptions, knowledge, preventions, and treatments of malaria.

### Data collection and selection of study participants

The distinctive geo-medical landscape in this region has made it a vital site for malaria-related studies, which can fill in the gap left by the limited amount of previous scholarship with such a focus. To assess and understand the dynamics of social representations of malaria, the semi-structured interviews, informal conversations, and participant observations were conducted in the Wayerema II neighbourhood of Sikasso in southern Mali.

The results of this study presented here are derived from data gathered through an ethnographic study, which was conducted by the lead or first author (B.S.) in May-August 2011 and March 2016. It included a 6-month ethnographic immersion in the study community, during which B.S. used interviews as well as participant observations. He also, kept communicating with some of the respondents using social media platforms like Facebook and WhatsApp. The research work focused on the local perceptions, social representations of malaria, and the therapeutic itineraries for healthcare among people in this community, in the health district of Sikasso.

Furthermore, different interview guides with open questions were developed and addressed to the key informants (patients, health workers, traditional therapists, and mothers). The number of these informants was selected using a convenient sampling approach. All interviews were conducted in Bambara, the national language, and in French. B.S. carried out a total of 60 interviews in 2011 (phase 1), and a different additional 10 in 2016 with only mothers, health workers, and traditional therapists (phase 2). Overall, he conducted a total of 70 semi-structured interviews involving fifteen health workers, fifteen pregnant women, twelve mothers, five heads of families, five community elders, ten youths, six traditional therapists, and two local authorities during the two phases (Table 1).

**Table 1:** Demographic information of the selected participant

Informants	Number	Age
Health workers	15	20–60
Pregnant women	15	18–45
Children’s mothers	12	18–60
Heads of family	5	25–60
Community elders	5	65–80
Youths	10	15–40
Traditional therapists	06	40–75
Local authorities	02	50–60

The participant observation was conducted to elucidate the questions of perceptions, representations, and practices. It was also made possible through immersive experiences in the health structures, along with other sub-communities in Wayerema II, processes of which were considered helpful. It allowed for better familiarization with the key actors and informants in order to better understand their mentality and perceptions. The first author kept field notes throughout the study period to capture significant events, interactions and moments.

A note for reflexion: “*I, B.S., include my own experiences as a Malian and a resident of Sikasso municipality. Growing up in Wayerema II, my family, friends, and I contracted malaria on many occasions. It is these experiences and the effects of my malaria on my life and that of my kin that sparked my early interest in social experiences of malaria. I don’t include my experiences as representative of all Wayerema residents nor as privileged information; rather I include my experiences as a participant observer to reinforce what my respondents are already reporting about malaria in their lives*”.

The collected data were reviewed by going through the entire corpus while noting the emerging themes. Upon the second time reading, the themes were re-organized thematically before interpretation. After that, the themes were respectively analysed in light of their correlations to the main research question [13].

### Data analysis

The semi-structured interviews were audio-recorded, transcribed, and translated from Bambara into French, then later into English by the lead author (B.S.). The transcripts were examined and analysed for items/themes relevant to our research objective. All transcripts were then imported into Microsoft Word 2010 Software.

An inductive anthropological approach has followed privileging the community’s views and using participant observation to better access the social representations and biomedical knowledge of the inhabitants of Wayerema II regarding malaria. Furthermore, the data were initially sorted by relevant themes before in-depth analysis. The first author (B.S.) also utilized the thematic analysis approach to code and categorize these qualitative data [14, 15].

For purely ethical reasons, the consent and approval of all informants were sought upstream (in advance), using proper explanations of the objectives and purposes of our study. We have ensured to keep the respondents anonymous, by assigning each respondent an identification number. The names of all our informants mentioned in this paper are pseudonyms. The ethical approval was obtained through the *Faculté des Lettres, Langues, Arts et Sciences Humaines (FLASH)*, Université de Bamako, Mali.

## Results

The results of the interviews (n = 70) present the social characteristics of participants of the sample groups for the semi-structured interviews. The participants included health workers (n = 15), with respectively 10 in CScom (*Centre de santé Communautaire, or* Community Health Centres), 02 in CSréf (*Centre de Santé de référence*, or Referral Health Centre), and 02 in the paediatric service in Regional Hospital of Sikasso; pregnant women (n = 15), who were interviewed either at the CScom or home after some primary connections built and also with the help of the first author B.S.’s fieldwork at the CScom; the heads of family (n = 05); the community elders (n = 05), with 1 imam, 1 Catholic priest, and a Chief of Neighbourhood included in this variant group; and—furthermore—youth (n = 10) with 05 males and 05 females aged between 15 and 40; mothers (n = 12) who were selected and inquired in order for knowing or understanding their opinions, and informing us more deeply about their sick children; Traditional therapist or healers (n = 06) including 03 women and 03 men, aged 40–75. They and 02 of their patients were interviewed in their house where a piece/room was managed for consultation. In addition, the young people (3 women and 2 men) were their patients and got involved during the participant observation among the traditional therapists (tradi-therapists). We stayed connected and interviewed them a few days later at their home. Lastly, the local authorities (n = 02), the first one was a mayor of the secondary town hall of Wayerema II, and another a municipal advisor for sanitation and hygiene at the main or central town hall of Sikasso. Participants were largely Muslims, a few were Christians and African spiritualists or “Animists”.

### Cultural and modern representations of malaria

For the interviewed residents of Wayerema II, the sociocultural conception of malaria is conveyed through the word *sumaya*. Their understandings of malaria are shaped through their experiences related to the disease, constructed through expressions and discourses surrounding it, and particularly influenced by existing religious, supernatural, and environmental factors. In this regard, Vamara, an elder, 70-year-old, told us: “*Sumaya is a very bad disease which killed our child. You see my son [His son here refers to B.S., he called him that because B.S. was the same age as his last-born child, and this is very common in Malian culture], sumaya is more problematic in the rainy season [June-October in Sikasso] because of our humid environment, and it is raining more in Sikasso region than others in Mali. So during this season we always have malaria*”.

*Sumaya* is of Bambara origin and refers to humidity and freshness in its literal sense (*suma* means freshness, coolness, humidity, and *ya* means situation in Bambara). The connotation herein is also related to the rainy season or winter highlighting the rain-triggered freshness from June to October. Therefore, the term itself is associated with coldness and rain/humidity as seasonal factors, corresponding to the period with very strong multiplication of *Anopheles gambiae*, the common type of mosquito in these areas.

With regard to the relevant symptoms, in an interview, Alice, a 23-year-old female student said, *“sumaya comes with vomiting, fever, chills, general stiffness, and kono. Dizziness is also a sign of sumaya.”*

Alice’s representations of malaria coincide with the understanding the lead author, B.S., had of the disease growing up in Sikasso. B.S. learned in his childhood that malarial conditions are accompanied by vertigo, vomiting, heightened body temperature, and anorexia, all interpreted by kin and neighbours as a manifestation of *sumaya*.

## Aetiologies of malaria: food, “malaria grain” and parasitic infection

The informants used the terms *sumaya* and *palu* interchangeably to describe the causes of malaria. In French, malaria is called *Paludisme*. *Palu* is an abbreviated form of the original French word used in communication on a daily basis. Residents of Wayerema II identified fruits and burning sun as one cause of malaria. According to Batoma, a 38-year-old pregnant woman diagnosed with malaria in the CScom (*Centre de santé Communautaire, or* Community Health Centres) of Wayerema II, “*the wounds or sore in the belly [kononandjoli in Bamanakan], the burning sun and fatty food are the causes of sumaya.*’’.

Likewise, another informant also attributed the causes of *sumaya* to food.

Nafissa, a 19-year-old female fruit seller said, “*Foods which are planted like bananas, eggs and unprotected foods (which have been exposed to open air) [are causes]. Also, oranges, mangoes, shea nuts are the causes of sumaya.*”

A respondent explained malaria through a concept of a “potential” or “dormant malaria” locally known as *sumaya banakise*.

Our respondent, Massita, a 38-year-old pregnant housewife and CScom (Community Health Centres) patient noted, “*The sumaya seed already exists in everyone’s body, especially in the rainy season or during the cold season. All it takes is a few triggers, such as oily and fatty foods, for the disease to break out*”.

Wayerema II residents also attributed *sumaya* to supernatural factors. The word *kono* emerged as a key term in explaining this causal factor. The interviewee, Mr. Zanga, a 70-year-old farmer explained, “*The disease of the kono is related to a malefactor bird. This demonic or evil bird flies over the roofs of houses during the twilight [fitiri] and puts an evil substance in the breasts of the nannies. When these nannies are going to breastfeed, they do it with their poisoned breasts. Then the evil bird will attack the soul of the children through manifestations of febrile convulsion. You can see the child caught by kono imitating the gestures of the flying bird. The child beats his arms against the ground, which usually leads to death*.”

Further, another excerpt from our observation illustrated very well how malaria is related to *kono*, and vice-versa in Zoumana’s family. Zoumana, a 48-year-old trader, had a little boy named Lassana, a 3-year-old, who had been ill for two days. On the morning of the third day after the first author (B.S.)’s arrival, Lassana’s father informed him that his wife and son were in the Cscom now. B.S. asked him how? Zoumana replied that the *kono* took his baby Lassana, and they took him to the traditional practitioner after putting water on his body and head by themselves. The traditional practitioner/therapist massaged him with some products and told them to bring him the next day. Unfortunately, Lassana lost consciousness this morning because of *kono* and they quickly took him to the CScom. He (B.S.) went to the CScom, and the little Lassana was hospitalized for very severe malaria. The medical doctor in chief of Cscom, Dr. Guindo told him Lassana was convulsed when they came here. (Participant observation, and informal conversation in the family of Zoumana, Wayerema II, June 2011).

The informants recognized the relations between the urban ecological environments which makes it an ideal breeding ground for mosquitoes. They also know that the root cause of malaria is the bite of *Anopheles gambiae* contaminated with the most common and dangerous pathogen in Africa and worldwide, the *Plasmodium falciparum*.

Mrs. Charlotte, a 43-year-old midwife working in a CScom, recognized the connection between mosquitoes and the urban environment of Wayerema II. She explained, *“Intra residential crops, grasses, stagnant water, wastewater, and non-existence of gutters, promote the multiplication of mosquitoes. Furthermore, the palu is caused by the bite of the infected female Anopheles.*”

Mr. Boubacar, a high school teacher, aged 41, concurred with Mrs. Charlotte, pointing out the relationship between malaria and the environment. Similar to her, Boubacar was very specific about the identity of the agent. He explained, “*The puddles of stagnant water, the wastewater of the neighbourhood are the places favourable to the development and the multiplication of mosquitoes. These cause the spread of malaria through the bite of the female anopheles. So the only cause of malaria is the bite of the infected female anopheles.*”

### Knowledge about signs and symptoms of malaria

The symptomatology of malaria in Wayerema II is multiple. Most respondents are aware of the clinical symptoms of malaria, including fever, yellowish vomiting, chills, general stiffness, *kono*, and vertigo. However, in their explanations, the narrative about biomedicine and biomedical methods has to some extent distinguished itself from the local and popular descriptions by confirming malarial diagnostics with the use of rapid diagnostic tests.

According to Mrs. Sanata, a 50-year-old CScom nurse, “*some of the malaria symptoms we see here are fever, headache, nausea, dizziness, joint weakness, abdominal pain, diarrhoea, and chills. Besides, our way to certify someone has malaria is the blood test.*”

Such was the case of Binke, a 64-year-old traditional therapist, who told us, “*The symptoms of sumaya are constipation, diarrhoea, headache, general fatigue, stomach pain, dizziness, vomiting, kono, and chills.*” Similar to Sanata, binke listed pain, chills, and diarrhoea as symptoms of malaria.

### Therapeutic routes: self-treatment

This section describes the steps the inhabitants of Wayerema II follow to treat malaria. One common therapeutic route is self-treatment, either before—or sometimes concurrently with—hospital treatment. For instance, the therapeutic route for Mamadou, which is presented next, involves self-medication as a first response, and only when it fails would he consult the CScom. One of the family members of Mamadou, a 65-year-old farmer, described to us how he treated *sumaya*:

*“Mamadou’s treatment starts with going to the small bush to cutting some leaves and tree roots like N’golobe, Sekoufali, Kosafune, and N’galama. Then his wife prepares these leaves and/or roots, and he drinks the decoction well done. He would not go to CScom unless these products cannot treat him or his family members”*.

The observation of Robert’s family involved the example of a 5-year-old boy. The boy’s name was Nicolas and he had mild signs of malaria. He had some clinical signs (fever, headache, and high body temperature), and he could not play with his cousins in their house. Little Nicholas’ grandmother, Alphonsine, a 70-year-old, observed his condition and talked with his mother. Then, grandmother, Alphonsine took care of her little boy by washing his body with *Tabakoumba* and *djala* roots, giving him papaya and *ngalama* decoctions, and massaging him with shea butter (*shitulu*) while reciting incantations (Participant observation of Robert’s family in Wayerema II, March 2016).

It was also found that, in addition to plants, the incantations cited for *shitulu* (butter/oil of *Butyrospermum parkii*) were used to treat malaria. This shea butter is a remedy for several kinds of disease in the study area and elsewhere in Mali. It can be used with or without incantations, *kilishi* in Bambara because it had therapeutic virtues in itself. As for the *kilishi*, it had a magico-religious and transformative or metamorphosing power. Applied when added to shea butter/oil, these magical formulas or *kilishi* treat the child when put on his body.

### Socioeconomic impacts of malaria

The effects of malaria on people are manifold, and the attention pay close to not only the medical but socioeconomic consequences on the lives of Wayerema II residents. *Sumaya* interferes with agricultural cycles and can lead to the shortage of labour needed for food production in Wayerema II, which still largely depends on agriculture. This is what Mr. Mamadou, a 65-year-old farmer, told us about its subsequent effects: “*The period when malaria prevails in our country also coincides with our production period of agriculture which is the rainy season. It was during this period that we left for the field. But last year the sumaya struck down my three young men and we did not have a good harvest. So it affected my family’s revenue*.”

Sagara is a 55-year-old repairman of *Jakarta* motorcycles shop living in Wayerema II. When he suffered from malaria, it was his wife who managed in hard conditions to support and take care of the family financially until her husband’s recovery. (Participant observation in Wayerema II, July 2011).

## Discussion

This ethnographic study explored the individual perceptions and socio-cultural representations of Wayerema II residents in Sikasso town in southern Mali regarding malaria, and how these conceptual understandings or explanatory narratives affect Wayerema II’s therapeutic routes. The results thematically are presenting and discussing below.

Respondents like Alice quoted above associate malaria with seasonal and environmental factors such as humidity and freshness produced during the heavy rainy season in central Mali. Associating malaria with seasonal and environmental factors suggests a miasmatic understanding of malaria. This finding corroborates other studies from sub-Saharan Africa that show a miasmatic understanding of the disease is part of the diverse disease explanatory framework used by Africans to make sense of their somatic and social conditions [7, 16–19]. However, in contrast to European theories of miasma that view disease as products of rotten matter and odour, the theory of Wayerema II is miasmatic insofar as it sees the environment as the cause of disease and not because of decomposition and the “bad air” it produces [20]. Wayerema II sees the freshness and humidity as the causes of the diseases and not necessarily the decomposition of matter and its derivatives.

However, in the same description of *sumaya*-related symptoms, Alice also mentioned *kono*, further complicating local cultural and social conceptions of malaria. In popular understanding and our respondents’ descriptions, *kono* refers to an “evil bird,” a supernatural and/or witchcraft-related cause of symptoms. Analysts can describe fever and dizziness as symptoms of *sumaya*, but for respondents in this study, *kono* operates both as a symptom; that is a sign of sumaya but also as a causative agent per se. In other words, *kono* is caused by a different agent, a supernatural force but not environmental or seasonal conditions—yet it is referenced when our subjects spoke about *sumaya*. In doing so, the informants of this study were suggesting both a miasmatic and supernatural layer of disease representation [7, 16, 20–22]. It appears the respondents sometimes mix *sumaya*, and *kono* together because *kono*’s symptoms, such as convulsions, resemble possessions or epilepsy, which is locally classified as a supernatural disease.

The participants of this ethnographic study did not always separate symptoms from aetiology in their understanding of malaria. This affirms the work of social scientists who have suggested that among African communities such as the Wayerema II, the interest is more about creating meaning and accessing the boundaries between the normal and the pathological [23, 24]. Does the shift from more somatic physical conditions to those in the mental or cognitive category demand a different explanation? This could be the case because communities view *sumaya* as seasonally and environmentally linked, while *kono* is more of a supernatural presence. The shift in representation of the disease could be a factor in how the disease progresses from one state to the other.

The term *kono* was not confined to the Wayerema II community. Other studies in Mali and Burkina Faso have documented the use of the term Kono, sometimes called “bird illness” used to describe convulsions among children [7, 16–18], and among the Wolof communities in Senegal where it is also called *sibiru*, meaning “warm body” [6, 24, 25]. *Kono* and *sibiru* are used interchangeably in these communities, including Wayerema II. Apart from the variations and linkages between *kono and sibiru*, in many African communities, malaria is attributed to “witch birds’’ or supernatural forces in the sky [21, 22, 26]. The inclusion of *kono* as a symptom or part of *sumaya*, judging from the former’s characteristics and intensity, indicates a severe form of malaria or even cerebral malaria. It could be widely acknowledged that if *sumaya* is a general representation of malaria or its milder forms, then *kono* refers to a more severe form of malaria such as cerebral malaria in the biomedical paradigm [16, 18].

But the explanation of sumaya as a general form of malaria and kono as a severe form of malaria is complicated by the respondents’ notion of sumaya banakise, which means the grain of malaria. As they (the respondents) noted in explaining that sumaya banakise “the body is in the state of expectation malaria”. This term seems to point to both an individual’s potential and vulnerability to contracting the disease and/or a latent state of malaria. It implies individuals have (variable) potential to contract malaria or arouse previously dormant malaria when one ingests acidic, sweet, and fatty foods that trigger the onset of the disease [7, 17].

While the participants’ explanation seems to refer to a grain of malaria as something related to the person’s immunity or the presence of *P. falciparum*, it does not foreclose the possibility that the presence of *sumaya banakise* is related to *kono* or other supernatural causes. The point is researchers cannot make a leap of interpretation that *sumaya banakise* is simply organic and not related to the supernatural [8, 19, 21]. Unravelling the local conceptions of malaria is complex and context-specific. It is not the purpose of this study to reconcile the different representations of malaria, nor to translate it into biomedical terms and theories, For example, while biomedicine demarcates nosography, aetiology, and diagnosis in separate containers, we are not entirely sure if our subjects do the same. The aim is to understand how residents of Wayerema II made sense of and explained malaria in their own terms and discourses.

## Aetiology of malaria

### Linkages between sumaya and food

In explaining the causes of sumaya, the respondents also identified dietary, environmental, and biomedical reasons. An intriguing suggestion from the respondents of this study was the explanation that *sumaya* can be caused by fatty food such as shea butter, fruits with acidic properties such as oranges, and sweet foods like wild fruits and corn. The respondents’ claims about the association between food and malaria were mostly general; however, they pointed to two intriguing ideas. The first idea is that “opened” sweet foods such as bananas and corn are more likely to cause malaria. If “open” here means how the banana or corn is preserved in opposition to “closed,” as in eating it fresh meaning never preserved, then this idea confirms existing studies about the association between food and diseases. Association of fatty, sweet, or acidic food to malaria is part of a growing discussion among Africans from different countries on changing lifestyles and their relations to food and in turn, how new dietary patterns create conditions for diseases like high blood pressure, diabetes, and sometimes cancer [7, 17]. In Julie Livingstone’s *Improving Medicine*, she writes that liver cancer is caused by the combination of “subclinical infections with hepatitis and aflatoxins in poorly stored African grain” [27]. While malaria is different from cancer, the participants’ claims about food as the causative agent for diseases collaborate African patients and health care providers’ discourse that links the two together [7].

Another related idea about the role of food in causing malaria connects back to *sumaya banakise*. As noted, the term seems to describe either a person’s potential to contract malaria or a form of dormant malaria. In addition to weakened immunity, our respondents suggested that “the grain of malaria” can also be triggered by the intake of fatty food. This does not necessarily mean the expression of *sumaya banakise* comes from a different condition than a compromized immune system. The interpretation of these explanations is to show that Wayerema II residents consider malaria from not a singular cause but multifactorial including the product of synergistic interactions between the type and state of food and its interactions with existing physiological conditions such as having the presence of the “malaria grain.”

### Biomedical explanations: causes and symptoms

If this study has created the impression that the residents of Wayerema II only conceptualize malaria through supernatural expressions that are not the case. Like in other African countries, Malians are living in a medically pluralistic society employing various traditions and cultures, such as biomedicine, to treat and explain their ailments [10, 28, 29]. Some informants explained the causes of malaria particularly through a biomedical lens [30, 31]. Both Wayerema II residents and healthcare workers or traditional therapists have an understanding of the clinical symptoms of malaria. The informants who practise traditional medicine also recognize the same symptoms as those diagnosed by biomedical practitioners such as fevers, shivering, headcheaches, vomiting, and body ache. Moreover, residents are also aware that mosquitoes cause malaria.

The data collected from health workers in the CScom of Wayerema II, Csréf (referral hospital of the city or referral health centre) of Sikasso, and paediatric services of Sikasso regional hospital pointed out that the nosographic entities (symptoms) appear mainly in rainy periods, which is from June to October. This period is also called the high malaria transmission season because of the factors that promote the multiplication of the disease’s pathogen. This observation lends credence to Wayerema II’s description of *sumaya* as a phenomenon related to “wetness” or the wet season. The difference between biomedical explanations and what we might call “indigenous” explanations is that the latter is multifactorial bringing in biomedical, spiritual, and environmental causes together in a specific manner.

Residents’ knowledge of symptoms and causes of malaria through a biomedical lens is in part fuelled by the efforts made by the Malian government in collaboration with its partners, such as the World Health Organization (WHO), United Nations Development Programme (UNDP), and media campaigns involving print, radio, and television industries. In fact, among those agencies working on traditional medicine such as the Department of Traditional Medicine, traditional therapists and herbalists have associations and collaborations with the National Malaria Control Programme (NMCP) to promote long-lasting insecticidal nets (LLINs), generic drugs, and free care for children under five and pregnant women in all Malian tiered health structures from regional hospitals to village dispensary [3, 18, 32]. In a way, Wayerema II’s residents’ rich accounts of malaria are micro-expression of larger state interventions to control malaria and shape peoples’ attitudes to malaria that often prioritize biomedical models to the subordination of local, situated responses and epistemologies [33].

### Sumaya and environmental causes

It is interesting to note that the respondents’ explanations of *sumaya* are connected with the environment such as seasonal variation. Mrs. Charlotte’s biomedical explanation quoted in the "Results" section also points to moisture or wetness as a site and occasion when malarial agents actively develop. Such views coincide with findings from some other studies that examine how African subjects interpret their disease in light of seasonal variations [7, 30, 31].

The indigenous explanation of aetiologies seems more diverse, combining environmental and supernatural factors, while the biomedical explanations focus on the agents and female anopheles as vectors. Nevertheless, some similarities in vectors can still be spotted between both explanations. First, in both accounts there is a vector present therein: for Wayerema II residents, it is the bird and for biomedical practitioners, the female *Anopheles gambiae*. In the indigenous explanation, *kono*, pernicious malaria, is caused by a witch bird, a kind of social poison in Wayerema II. In the biomedical realm, the vector is the mosquito, but the “poison” is *P. falciparum.* Again, it is not implying that biomedical terms equate to indigenous systems, which are various per se, but pointing to some intriguing parallels and perhaps syncretism, as in a mixture of ideas, paradigms, and theories that are both indigenous and more or less “western” that have percolated in these communities over many decades. This qualitative study cannot ignore that biomedical practice in Africa has lasted for over one hundred years and therefore cross-fertilization of ideas has been happening in a bidirectional way in urban areas of Mali [31, 34].

### Therapeutic routes

The study was interested in linking how our respondents understand and interpret malaria through drawing from their social and cultural understanding and how such conceptions influence their therapeutic resources [5, 6, 8–10, 19, 21, 26, 28, 35, 36]. Arthur Kleinman’s notion of therapeutic routes instructive in explaining and understanding the process and decisions-making inherent in the respondent’s health seeking-behaviour. Arthur Kleinman has theorized that a therapeutic route comprises three stages, and it begins with the subject’s sensing a malady and his/her inclination to interpret it within the frame of illness per se. Such illness perception is what it refers to as the first stage of a malady (illness), where an individual feels a sense of imbalance or anomaly that might be linked to a certain name of the illness. This would give rise to the second stage: the subject communicates with a healthcare provider, healer, or kin about his/her experience, which may lead to the further recognition of the malady. This process allows the subject to justify his/her role as a patient by having the abnormal state (or sickness) socially recognized. Furthermore, the third stage involves the act of defining an illness in nosographic terms, which enters the biomedical dimension and is grounded upon the nomenclature of the sick state by the therapist [37, 38].

This study also revealed that the practice of self-medication is more often the first resort in case of sickness manifestations such as headache, fever, and dizziness. Considering the case of Mamadou that we recounted in the "Results" section, he used plants and herbs for self-medication often. Mamadou, moreover, described it as “the easiest thing in the world.” By this, he means self-medication can be an everyday practice. It also signifies that he has the knowledge of plants as cures and can by himself distinguish which are easily available and relatively inexpensive for use. The Wayerema II residents usually use the decoction of “bitter” plants such as *Combretum Micrathum* (*golèbè*), *Vernonia Colorata Will* (kosafunè), and *Anoeissus leiocarpus* (n’galama). Mamadou case and others reinforce the observation that upon the appearance of a disease, the first response takes place at home and draws from situated knowledge of healing. Thereafter other routes follow not necessarily in linear order as in starting with a traditional healer and then a biomedical doctor.

Self-medication, as the first therapeutic recourse to deal with ailments like malaria, fever, headaches, and other diseases, has been reported by other authors in African and Asian contexts [6, 8, 17–19, 33, 35, 39–43]. There are some minor differences in details with this therapeutic recourse given the prevailing conditions. It was found that when a person is sick, especially with signs of a complicated case of malaria, he/she would consult family or a local therapist. These remedies used are various but generally can be classified into three types: (1) the traditional sector comprised of traditional healers, herbalists, or *marabouts* (Muslim healers), (2) the formal sector, which includes hospitals, community health centres, CSréf, private hospitals, and (3) the informal sector, such as street vendors, drug peddlers, and the health workers practicing “privately.” These practices are the main routes that the Wayerema II residents use to heal, in line with the results of previous studies [9, 21, 23, 43]. They are also the main factors in cases of severe malaria and cases of pre-transfer in the community health centre (CScom) of Wayerema II.

It can also add that plants used for self-treatment are often “dose-free’’ and can be “effective’’ against malaria. Often people would only go to the community health centre or a health facility after the disease worsens or approaches towards *kono*. Sometimes, malaria cases brought to the CScom are more severe, but also better-treated. Indeed, the agents of CScom must evacuate the patient(s) to the CSréf or regional hospital of Sikasso. In the meantime, self-medication can be “modern.” Modern self-medication is practised in Wayerema II using anti-malarial pharmaceutical products, such as Maloxine, Madar, Quarcitem, and Co-arinate. Self-medication is also done with anti-malarials from street vendors of illicit medicine or “pharmacies par terre,’’ literally meaning “pharmacies on the ground”, with these anti-malarials being “counterfeit.’’ These products contain “fatokèni’’ (*sudrex*), “sampinrin” (*ibumole*), and “bérébila” (*ipucup*), which mean respectively in English, the “little fool,” the “lightning,” and the “leave the stick and get on the feet quickly.” These designations and terms are not accidental because they reflect the effects of these “fake drugs’’ on people. These products are often used because of the high cost of malaria treatment which is unaffordable for certain social groups, especially the underprivileged ones. The treatment of mild malaria costs about 5–8 USD, while treatment for a complicated case costs about 18–27.5 USD [36, 44–46]. Probably linked to financial affordability, these participant observations in Wayerema II and other studies have established that self-medication is the main therapeutic route for Wayerema II populations and other African communities in Western Africa such as Nigeria, Senegal, Burkina Faso, and Côte d’Ivoire [7, 19, 25].

### Sumaya and socio-economic impacts

Another socio-economic consequence of malaria is absenteeism and loss of productivity, especially for farmers and people involved in informal sectors. Many Malian families (nuclear, limited, or extended) depend on income generated by a single person who fulfills family needs such as the allocation of food, education, and health. For example, if the latter falls ill to *sumaya*, the social, economic, and psychological burden would fall onto the whole family. Mamadou, whose story was recounted above, used the term “three valid arms” to describe the sense of powerlessness and precarity when three of his sons got sick from malaria and therefore, were unable to help with farming obligations. The term both captures the level of impact it has on her as a parent, but personally: their sickness signified the loss of her own body parts. This term lays bare the serious impact of malaria on the whole family, even if merely one individual gets sick. The situation is dire for people who depend on farming, like Mamadou. The affliction of her three “valid arms” put her food and financial plans in a precarious situation.

This study coincides with some other studies conducted in sub-Saharan contexts that show the socio-economic impact of malaria at the family level and the ways in which it shapes therapeutic recourse [25, 36, 46]. First, the financial cost of treatment is very high and modest heads of families often prefer to take care of themselves at home or pay for drugs from alternative vendors. As such, intra-household self-treatment or intra-domiciliary self-treatment saves time and money for parents. The income per capita for Malians is 2.5 USD per day in 2020 according to the World Bank. Treatment of simple malaria costs 4–8 USD, and for treatment of severe malaria, the number rises to 25–35 [3, 36, 45]. The cost of simple treatment is twice the daily earnings and for complicated malaria almost one fifth of monthly earnings.

## Limitations

In our study, a qualitative ethnographic approach was developed with random sampling. This study has a limitation in that it does not represent the perceptions and social representations of all communities and health professionals in the District of Sikasso. As information was collected only using anthropological methods and tools, triangulation was unnecessary and could not be done, the study’s limitation also. Nevertheless, to the authors’ knowledge, this is one of the first studies conducted in the urban endemic malaria area in Mali to explore and examine the social representations, therapeutics routes, and treatments of people regarding malaria.

## Conclusion

The finding of this study shows that the residents of Wayerema II in the health district of Sikasso have complex knowledge of the aetiologies, and the nosographic entities of malaria, its seriousness, and preventive measures against the vector or pathogenic agent. Wayerema II residents’ conception of malaria did not amount to a unified “indigenous” explanatory model nor did it neatly fit the biomedical paradigm. Fundamentally, integrating local and popular knowledge of malaria into the biomedical registry must be a priority for understanding the knowledge itself, and the attitudes, beliefs as well as practices associated with it, which significantly shape ways of prevention and treatment. One possible implication of this knowledge is to recruit traditional healers in referral to formal health centres and involvement in malaria health campaigns that draw out the merits of traditional and biomedical paradigms.

Furthermore, this study has shown how much the anthropological data, in an urban context, carry to (re-)consider in an understanding of the choice of therapeutic routes and health seeking-behaviour adopted, which provide insights into the development of the programme, and new strategies fighting against malaria in Africa in general, and in Mali in particular.

## Data Availability

The datasets during and/or analysed during the current study are available from the corresponding author on reasonable request.

## Abbreviations

SLIS: Système Local d’information Sanitaire
CSCom: Centre de Santé Communautaire (Community Health Centre)
WHO: World Health Organization
UNDP: United Nations Development Programme
NMCP: National Malaria Control Programme
LLITN: Long-Lasting Insecticide-Treated Net
CSréf: Centre de Santé de référence (Referral Health Centre)
ACT: Artemisinin-based Combination Therapies
